# Effects of bed compression on protein separation on gel filtration chromatography at bench and pilot scale

**DOI:** 10.1002/jctb.5411

**Published:** 2017-10-09

**Authors:** Darryl YC Kong, Spyridon Gerontas, Ross A McCluckie, Martin Mewies, David Gruber, Nigel J Titchener‐Hooker

**Affiliations:** ^1^ Department of Biochemical Engineering University College London UK; ^2^ Ipsen Limited Wrexham UK

**Keywords:** bed compression, size exclusion chromatography, Sepharose CL‐6B, HETP, preparative chromatography, protein separation

## Abstract

**BACKGROUND:**

Poorly packed chromatography columns are known to reduce drastically the column efficiency and produce broader peaks. Controlled bed compression has been suggested to be a useful approach for solving this problem. Here the relationship between column efficiency and resolution of protein separation are examined when preparative chromatography media were compressed using mechanical and hydrodynamic methods. Sepharose CL‐6B, an agarose based size exclusion media was examined at bench and pilot scale. The asymmetry and height equivalent of a theoretical plate (HETP) was determined by using 2% v/v acetone, whereas the void volume and intraparticle porosity (ϵ_p_) were estimated by using blue dextran. A protein mixture of ovalbumin (chicken), bovine serum albumin (BSA) and γ'‐ globulin (bovine) with molecular weights of 44, 67 and 158 kDa, respectively, were used as a ‘model’ separation challenge.

**RESULTS:**

Mechanical compression achieved a reduction in plate height for the column with a concomitant improvement in asymmetry. Furthermore, the theoretical plate height decreased significantly with mechanical compression resulting in a 40% improvement in purity compared with uncompressed columns at the most extreme conditions of compression used.

**CONCLUSION:**

The results suggest that the mechanical bed compression of Sepharose CL‐6B can be used to improve the resolution of protein separation. © 2017 The Authors. *Journal of Chemical Technology & Biotechnology* published by John Wiley & Sons Ltd on behalf of Society of Chemical Industry.

## INTRODUCTION

Chromatography is the workhorse of the bioprocessing industry where it is utilised primarily for the separation of target molecules from impurities. This is achieved by passing a mobile phase, which contains both product and impurities, over a stationary phase; the properties of the stationary phase impart selectivity that results in separation of the target molecule from its impurities. Modifications to the stationary phase can result in selectivity based on size (size exclusion), charge (ion exchange) and hydrophobicity (hydrophobic interaction). The stationary phase can be packed into a variety of formats, the most common of these being cylindrical columns. Size exclusion chromatography (SEC) separates a mixture of molecules according to their size. Smaller molecules diffuse into the stationary phase and hence are retarded through the column compared with molecules of intermediate size, which flow through the void volume in the column. Poorly packed chromatography columns cause uneven flow within the packed bed, this leads to zone mixing, band broadening and ultimately loss of resolving power which can impact the purity and yield of product.

Currently, the use of hydrodynamic flow is often the method of choice for packing size exclusion.^1^ However, there are few reports detailing the effect of different procedures of bed compression, i.e. mechanical compression, on packed polymeric particles.^2^, ^3^ At high flow rates bed compression occurs with a concomitant decrease in column permeability.^4^ Particles follow the direction of flow during column packing and become compressed at the column outlet. The voidage between resin particles is reduced with increasing flow rate, which can deform the particles.^3^ Furthermore, it has also been found that the voidage differs between the top and bottom regions of the column, suggesting that the column is not optimally packed.^5^ This highlights an opportunity to further compress these regions with large voidage towards the top of the column to create a more uniform packed column. As a result, channelling is reduced and this increases the accessible surface area for mass transfer within the column.^4^, ^6^

Highly cross‐linked, polymeric stationary phases with wide protein separation ranges are available for protein separation by SEC. Effective bed compression has been shown to reduce both the time and column size required to achieve optimum separation.^7^, ^8^ The particles of Sepharose CL‐6B are made of agarose, thus they are porous and mechanically soft. Agarose‐based beads are less stable than silica beads due to solvation of the polymer which leads to swelling and thus softening of the support and an inability to withstand high pressures.^4^, ^9^, ^10^

At moderate packing pressures, several investigators have observed that bed compression reduces interstitial porosity, which increases resolution and therefore favours column efficiency.^4^, ^11^ Stickel and Fotopoulos investigated the impact of a reduction in void volume, created during hydrodynamic compression, on column efficiency.^12^ Their results were interpreted using the Blake‐Kozeny equation, which correlates bed porosity as a function of linear velocity.^12^ By predicting the impact of operating parameters at industrial scale they were able to identify the most favourable conditions for column efficiency.

Meyer and Hartwick investigated the relationship between column efficiency and packing pressure for narrow‐bore columns with siliceous stationary phases and identified the existence of an optimum column packing pressure.^13^ However, no evidence based explanations were reported on intraparticle porosity. Though much is known about the hydrodynamic effects of compression on gel filtration beads; no theory is available to account for effects created by mechanical compression during scale up. The aim of this study was to characterize the relationship between the methods of column packing and column efficiency by applying hydrodynamic and mechanical methods of compression. This was achieved by using a commercially available gel filtration media, Sepharose CL‐6B (GE Healthcare, Uppsala, Sweden) to exploit any benefits that may accrue by compression for the separation of macromolecular therapeutics by size exclusion.^4^

## MATERIALS AND METHODS

### Bench‐scale setup

Bench‐scale experiments were carried out using the ÄKTA Avant 25 (GE Healthcare, Little Chalfont, Buckinghamshire, UK) fast protein liquid chromatography system equipped with pump unit P‐903, UV cell (280 nm, 2 mm path length), conductivity cell, and auto sampler A‐900. The control software UNICORN 6.0 (GE Healthcare, Little Chalfont, Buckinghamshire, UK) was used. The extra column dead volume was kept to a minimum by using 0.12 mm I.D. capillary tube to connect the column to the injector. An XK16 column (GE Healthcare, Uppsala, Sweden) was used with an inner diameter (I.D.) of 0.016 m (XK16, with adjustable column lengths). All chromatography experiments were performed in triplicate and at room temperature 20 ± 5 °C.

### Pilot‐scale setup

Pilot‐scale experiments were carried out using the ÄKTApilot system (GE Healthcare, Little Chalfont, Buckinghamshire, UK) equipped with pump unit P‐907, UV cell (280 nm), conductivity cell, and auto sampler A‐950 supplied with the UNICORN 5.11 control software. A BPG‐100/500 (GE Healthcare Uppsala, Sweden) was used with an I.D. of 0.1 m with adjustable column lengths. All chromatography experiments were performed in triplicate and at room temperature 20 ± 5 °C.

### Stationary phase and loading samples

Studies were carried out using a gel filtration resin; Sepharose CL‐6B (GE Healthcare Uppsala, Sweden). It is a 6% cross‐linked agarose gel filtration based matrix which may be used to separate samples of diverse molecular weight; 1 × 10^4^–1 × 10^6^ Da. The resin is available in both Sepharose and Sepharose CL forms where the cross‐linked form is chemically and physically more resistant, allowing identical selectivity but at increased flow conditions. The spherical resins had a size distribution of 45–165 µm (quoted by the manufacturer). The average bead diameter was determined to be, *d*_*p*_ = 98 µm ± 5 µm (Malvern Mastersizer 3000 laser sizer; Malvern Instruments, Worcestershire, UK).

All reagents were from a single supplier (Sigma–Aldrich, Poole, Dorset, UK) unless stated otherwise. The loading materials for this study were ovalbumin from chicken, BSA and γ'‐ globulin with molecular weights of 44, 67 and 158 kDa. A loading volume of 0.05 CV of 5 mg mL^‐1^ of total protein was used. The packing buffer used was a 20 mmol L^‐1^ sodium phosphate buffered saline (PBS) with 130 mmol L^‐1^ NaCl at pH 7.2. All samples were filtered using 0.22 µm Stericup filter units (Merck & Co., Darmstadt, Germany).

#### Bed compression procedure

Sepharose CL‐6B resin was made up to 80% (w/v) slurry in a 50 mL measuring cylinder. The total slurry volume was calculated based on achieving a desired bed height of 20 cm. Each bed was initially gravity settled overnight before flow packing at a velocity of 30 cm h^‐1^ (1.0 mL min^‐1^ for bench scale column) for 5 column volumes (CV). Once flow packed at this flow rate, a constant initial bed height of 20 cm ± 0.1 cm was achieved. A linear velocity of 30 cm h^‐1^ was applied during HETP and protein separation testing. Subsequently, bed properties were measured by asymmetry and height equivalent of a theoretical plate (HETP) to measure the impact of the methods of compression and the level of compression achieved. Two methods of compression were examined: hydrodynamic and mechanical compression.

#### Bed compression factor

As a consequence of each incremental increase in compression, bed height reduced. This was captured through the bed compression factor (λ) defined as:

(1) $$\lambda =\frac{V_{\mathrm{co}}-V_{c}}{V_{\mathrm{co}}}$$

where *V*_*c*_ is the packed bed volume and *V*_*co*_ is the initial settled bed volume. A maximum level of bed compression factor of 0.15 was used. This was well below the maximum pressure drop of 0.045 MPa, provided by the manufacturer. For both methods of compression, three repeats were conducted.

#### Hydrodynamic compression

For hydrodynamic compression, packing buffer was pumped through the column at the maximum flow rate of 150 cm h^‐1^ (5.0 mL min^‐1^ ‐ within the pressure drop limit) for bench scale column until the desired compressed bed height was achieved. Once the measured pressure drop (less than 0.036 MPa) remained constant for 1 CV, the top column adapter was immediately lowered to the matrix bed surface to retain the level of compression.

#### Mechanical compression

For mechanical compression, the top adapter was physically pushed down until the desired bed compression had been achieved. When lowering the top adapter, the O‐ring was loosened and the column inlet connector disconnected from the ÄKTA. This allowed buffer to escape at the top of the column during compression. Once compressed, the column adapter was secured and connected back to the ÄKTA. Care was taken to ensure no air was trapped in the tubing or column.

#### Methods of compression

Two methods of resin packing were investigated. The first method applied compression in a single step by packing the column from the original packed bed to the compressed state. This is referred to as one step compression.

1. Compression was applied to the bed in a single step until the desired bed compression factor was achieved by hydrodynamic or mechanical compression, described in the previous two subsections.
2. Compression was applied at four different compression factors (0.02, 0.05, 0.10 and 0.15).
3. Column was repacked for the next compression factor.

The second method went from the original packed bed to the compressed state by applying multiple series of steps. This is referred to as multiple incremental step compression.

1. For hydrodynamic compression, a flow rate of 30 cm h^‐1^ was applied and increased to 150 cm h^‐1^ until the desired bed compression factor was achieved. Mechanical compression was applied as described in the subsection ‘Mechanical compression’.
2. Four different compression factors (0.02, 0.05, 0.10 and 0.15) were applied starting with the lowest compression factor. The next compression factor was carried out without repacking the column.

When no compression was applied (compression factor of 0.00), the column was flow packed at a constant linear velocity of 30 cm h^‐1^ for 5 CV for both bench and pilot scale experiments as described in the section ‘Bed compression procedure’.

#### Process description

An equilibration step of 3 CV of PBS at pH 7.2 was used before loading the sample directly onto the column. A loading volume of 0.5 CV of 5 mg mL^‐1^ of total protein was used. Eluate fractions were collected until the UV trace returned to the baseline. A wash step of 2 CV was used to remove any remaining traces of sample. Following elution, the column was cleaned with 2 CV of 0.5 mol L^‐1^ NaCl and 0.1 mol L^‐1^ NaOH solution and then washed with ultrapure water (typically at 18.2 MΩ cm at 25 °C) until neutral pH was reached. Columns were stored in 20% v/v ethanol solution, as per the manufacturer's recommendations. Columns stored in 20% v/v ethanol were washed with 5 CV of packing buffer prior the equilibration step. Columns stored in 20% v/v ethanol were washed with 5 CV of packing buffer prior the equilibration step.

### Measurement of column efficiency, intraparticle porosity and protein separation

The data required for estimation of the quality of column packing was recorded using UNICORN 6.0 software. The reduced plate height and asymmetry were based on the axial dispersion of an acetone pulse. Acetone and dextran were used to assess the intraparticle porosity.

#### Acetone test

Column efficiency was measured by asymmetry and reduced plate height using a 2% CV injection of 2% v/v acetone, applied using a V‐7 sample injector with a 100 µL loop for the bench scale column and directly injected using the sample pump for the pilot scale column.

#### Blue dextran test

The voidage at each compression level was measured by an excluded tracer (blue dextran). Dextran is a glucose polymer with covalently attached reactive blue dye molecules of molecular weight 2×10^3^ kDa. The volume in which the dextran elutes represents the void space between the resin particles. The intraparticle porosity was determined by the elution profiles of acetone and blue dextran. The intraparticle porosity, ϵ_*p*_ is defined as:

(2) $$\epsilon_{p}=\frac{{\mathrm{EV}}_{d}-{\mathrm{EV}}_{a}}{{\mathrm{EV}}_{a}}$$

where *EV*_*d*_ is the elution volume of dextran and *EV*_*a*_ is the elution volume of acetone.

#### HPLC‐SEC protein mixture

SEC‐HPLC was used to determine the purity of the eluting protein mixture, this was performed using an Agilent 1100 HPLC system with ChemStation software and an Agilent ZORBAX GF T‐250 column (Agilent Technologies UK Ltd, Berkshire, UK). The total protein concentration was determined using the Bradford method with Brilliant Blue G Protein Assay reagent (Sigma–Aldrich, St. Louis, MO, USA).^14^ Gel filtration standards (Bio‐Rad Laboratories Ltd, Hertfordshire, UK) were used to calibrate the accuracy of the SEC‐HPLC column (data not shown).

#### Purification factor

Based on the SEC‐HPLC data collected from the flowthrough fractions, the separation performance was evaluated based on the purification factor (PF). The impurities in the sample load chosen to be ovalbumin (44 kDa) and γ'‐ globulin (158 kDa), whereas BSA (67 kDa) was selected as the product, to allow for separation of smaller and larger impurities. The PF is described as the ratio between the final purity of BSA after purification to the initial purity of the sample load.^15^

## RESULTS AND DISCUSSION

### Column efficiency tests

**Figure 1:**
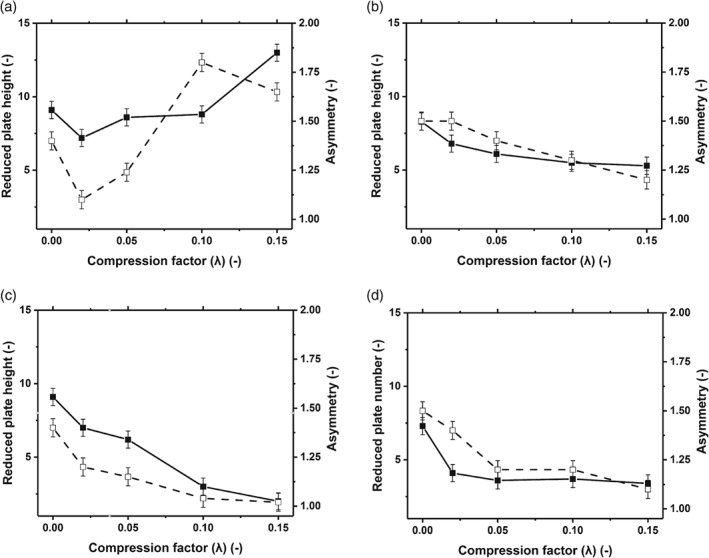
Comparison of reduced plate number and asymmetry for compressed beds achieved by hydrodynamic and mechanical methods. Columns packed with Sepharose CL‐6B 0.016 m I.D. 20 cm bed height. (a) Hydrodynamic compression achieved by multiple incremental steps (b) hydrodynamic one step compression. (c) Mechanical compression achieved by multiple incremental steps (d) mechanical one step compression. (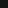) Reduced plate height; (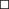) asymmetry.

The impact of two different methods of compression, hydrodynamic and mechanical, on reduced plate height and asymmetry were investigated. For each method of compression, four different compression factors (0.02–0.15) were achieved by multiple incremental steps or one step compression as presented in Fig. 1(a)–(d). It has been shown that a highly compacted region near the base of the column forms when hydrodynamic compression is used, where pressure will be the greatest.^5^ It appears that Sepharose CL‐6B achieved improved asymmetry and reduced plate number at 0.02 compression factor via hydrodynamic multiple incremental compression steps; however, the column efficiency declined as further pressure was applied due to the flow of buffer. This finding is consistent with literature.^3^, ^16^, ^17^, ^18^ The effect of hydrodynamic compression has been shown to cause flow instability and an increase in the reduced plate height, which detrimentally affects column efficiency.^16^ Mechanical compression yielded higher column efficiency than did hydrodynamic compression – a 3.5‐fold improvement in reduced plate height (Fig. 1(c)–(d)).

**Figure 2:**
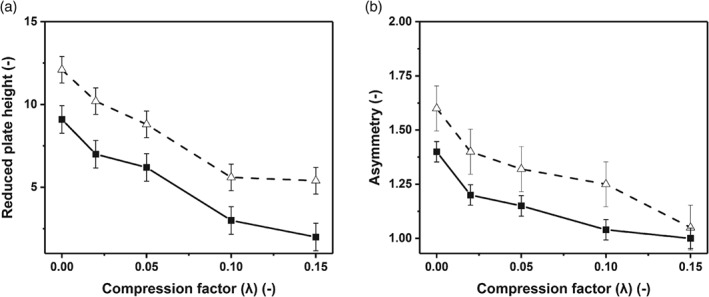
Comparison of reduced plate number and asymmetry achieved by mechanical compression defined by multiple incremental steps. Column was packed with Sepharose CL‐6B 0.016 m I.D. 20 cm bed height. Measurements were made using 5 mg mL^‐1^ BSA and 2% v/v acetone. (a) Reduced plate height comparison: (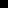) acetone; (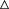) BSA. (b) Asymmetry comparison: (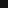) acetone; (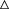) BSA.

Since mechanical compression gave better column efficiency, the impact of mechanical compression on asymmetry and reduced plate height was examined using BSA as a model protein. The impact of mechanical compression defined by multiple incremental steps are presented in Fig. 2(a) and (b). The reduced plate height improved at increased levels of mechanical compression. The improvement doubled as the compression factor increased from 0.0 to 0.15 (Fig. 2). Additional tests were performed to determine how mechanical compression influenced both the intraparticle and interparticle voidage (Table 1).

**Table 1:** Impact of mechanical compression achieved by multiple incremental steps on measured intraparticle porosity and bed voidage. Results obtained from the dextran blue and acetone elution profile data with Sepharose CL‐6B. Measurements were repeated three times with a relative standard deviation of less than 5% in all measurements

Mechanical incremental steps compression		
Compression factor	Intraparticle porosity	Voidage space
(λ)	(*ϵ*_*p*_)	(*ϵ*)
0.00	0.63	0.41
0.02	0.60	0.39
0.05	0.63	0.36
0.10	0.62	0.33
0.15	0.60	0.31

Mechanical compression led to a decrease in voidage but no discernible effect on intraparticle porosity. The voidage data is consistent with earlier work.^19^ It is believed that pore diffusion is enhanced as voidage within the column falls.^20^ This allows for greater surface area for diffusion between the resins and analytes to be presented to the molecules.^21^, ^22^ The consistent intraparticle porosity even under significant levels of mechanical compression may be explained by considering the elastic properties of the agarose material. Porosity moved from about 0.4 at no compression to 0.3 at a compression factor of 0.15 with mechanical compression. A porosity of 0.4 is expected with randomly packed spheres under gravity settling.^20^ When hydrodynamic compression is applied, stress on the stationary phase accumulates in the direction of flow indicating greater compaction at the outlet of the column. In addition, different regions of voidage space, particularly at the top of the column, result in uneven flow distribution when hydrodynamic compression is applied. By contrast, under mechanical compression, pressure is applied to the entire cross‐section at the top of the bed. This gives an opportunity to compress further the top regions with larger voidage to create a more uniform packed bed along the length of the column. This allows for a more even distribution of pressure along the length of the column when mechanical compression is used compared with hydrodynamic compression. In addition, near‐wall packing may be a possible source of poor performance under hydrodynamic compression, since uneven pressure across the cross‐section may cause uneven velocity distribution, particularly at higher flow rates.

Others have reported that the void fraction is lower near the column wall than in central and upper regions of the column.^5^ These insights were gained using static magnetic resonance imaging (MRI) and were explained by the additional downward force on the upper regions of the column caused by movement of the top adapter which imposed mechanical compression on the bed.^3^, ^5^

Our study suggests that compression achieved by applying pressure through the movement of the top adapter results in a better quality of packing. The fact that the voidage decreases means that interparticle distances are getting smaller and hence mass transfer is expected to rise. The impact of improved mass transfer rates on adsorptive separation was outside the scope of the study but might provide a beneficial impact of column compression by improving separation time and/or the resolution achieved for a given bed.

### Effect of mechanical compression on protein separation at bench scale

**Figure 3:**
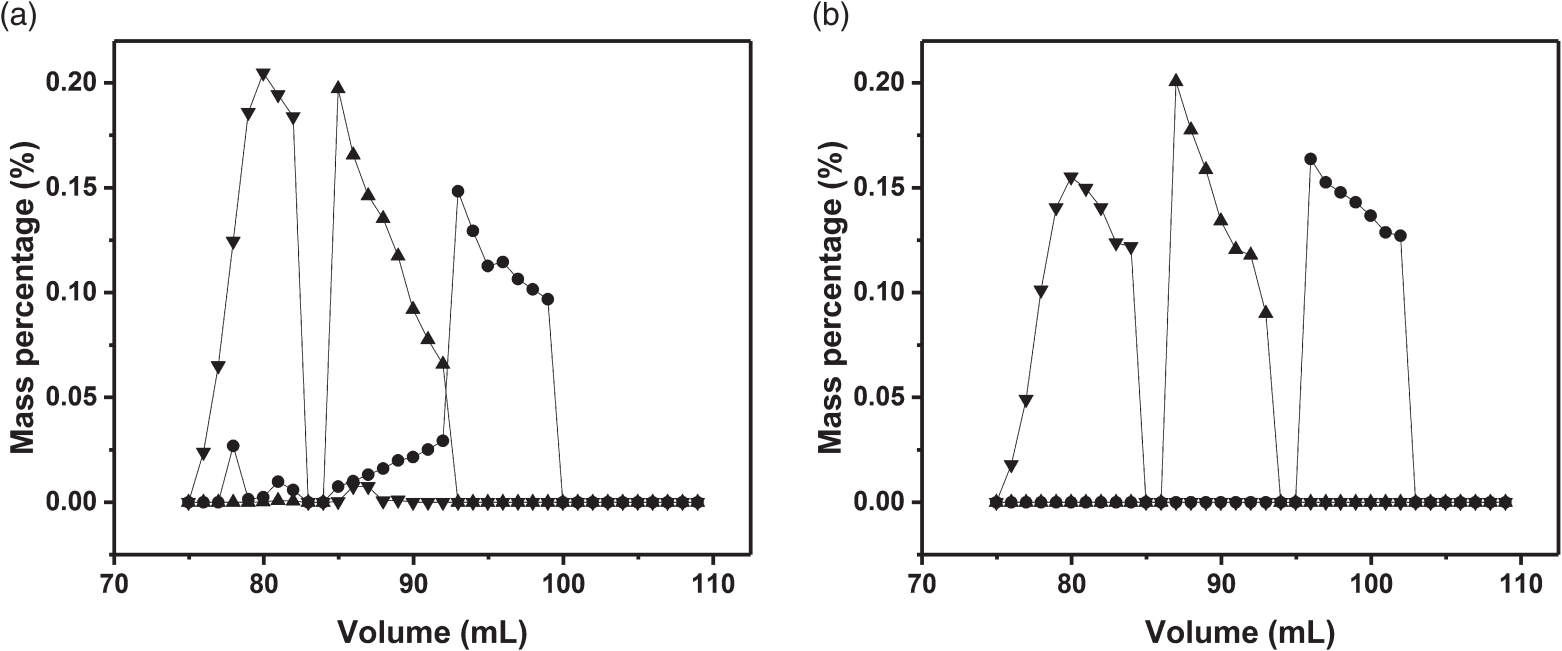
Impact of mechanical compression achieved by multiple incremental steps on the purity of a simple protein mixture. Mass percentage of each fraction based on HPLC‐SEC with a protein mixture of ovalbumin, BSA and γ‐globulin. (a) 0.0 mechanical compression; (b) 0.15 mechanical compression. (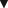) y‐globulin; (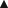) BSA; (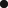) ovalbumin.

Table 2 summarises the impact of mechanical compression on the separating performance of the bench scale chromatography system. Figure 3(a) and (b) provide a simple schematic of the separation performance analysed at two extremes; 0.00 and 0.15 compression factor. Mechanical compression beyond a compression factor of 0.10 provided baseline resolution with improvements in both asymmetry and reduced plate height as well as greater peak resolution. These results indicate that for size exclusion separations the performance of a given protein separation can be improved by operating beds under mechanically compressed conditions compared with hydrodynamic compression at 30 cm h^‐1^. This is in contrast to earlier findings based upon hydrodynamic compression and suggests that the mode of compression is closely related to the column efficiency achieved.

**Table 2:** Impact of mechanical incremental steps compression on the peak resolutions directly measured by absorbance at 280 nm from the resulting ÄKTA chromatogram. Protein mixture of Ovalbumin, BSA and γ‐globulin for Sepharose CL‐6B 0.016 m I.D. Measurements were repeated three times with a relative standard deviation of less than 5% in all measurements

Compression factor (λ)	Bed height (cm)	Resolution peak 1 and 2	Resolution peak 2 and 3
0.00	20	0.9	0.9
0.02	19.6	1.2	1.1
0.05	19	1.5	1.3
0.10	18	1.6	1.5
0.15	17	1.7	1.8

**Figure 4:**
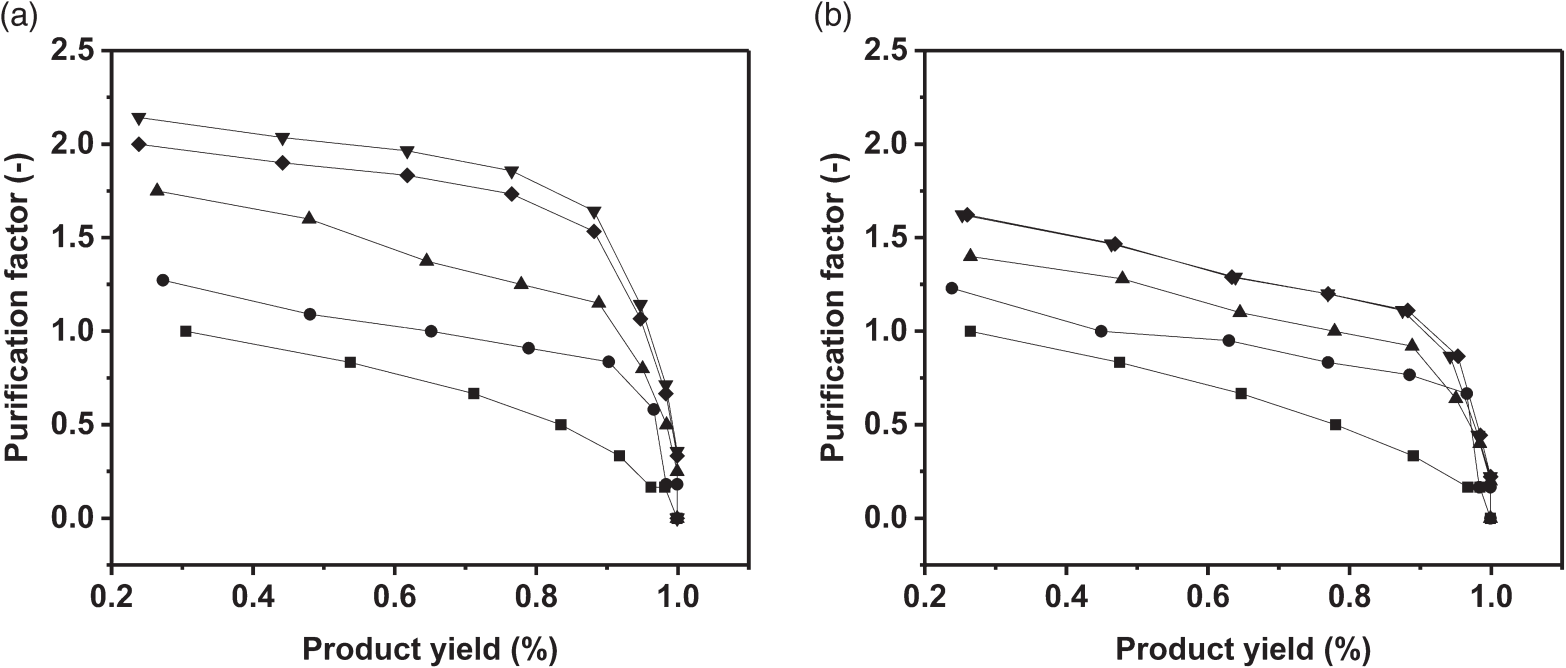
Impact of mechanical compression on separation performance of a fixed protein mixture. Purification factor vs product yield of a protein mixture of 5 mg mL^‐1^ with mechanical compression at bench scale. (a) Mechanical incremental steps compression; (b) one step mechanical compression. (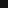) 0.0; (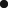) 0.02; (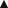) 0.05; (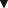) 0.10; (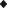) 0.15.

Figure 4 displays the purification factor as a function of yield for separating a fixed protein mixture obtained with columns that had undergone mechanical compression. Results show that mechanical compression via multiple incremental steps leads to greater levels of product purity and yields than mechanical compression in one step. The results indicate that performance of protein separation is better the higher the level of mechanical compression achieved, but that compression by multiple incremental step protocols created separation with significantly higher purification factor (PF) values for all yields. This was especially pronounced for compression levels >0.05. For example, at a typical specification of product yield of 0.9 the PF at 0.15 compression was 1.25 for mechanical compression achieved in one step and 1.75 for mechanical compression in multiple incremental steps. Such increased PF offers the ability to increase purity at a set yield target or to increase yield with no detrimental impact on purity. Since mechanical compression in multiple incremental steps created separation with significantly higher PF values compared with one step compression, we consequently set out next to examine the impact of multiple incremental steps during scale up.

### Scale‐up comparison using XK16 and BPG100 with mechanical compression

**Figure 5:**
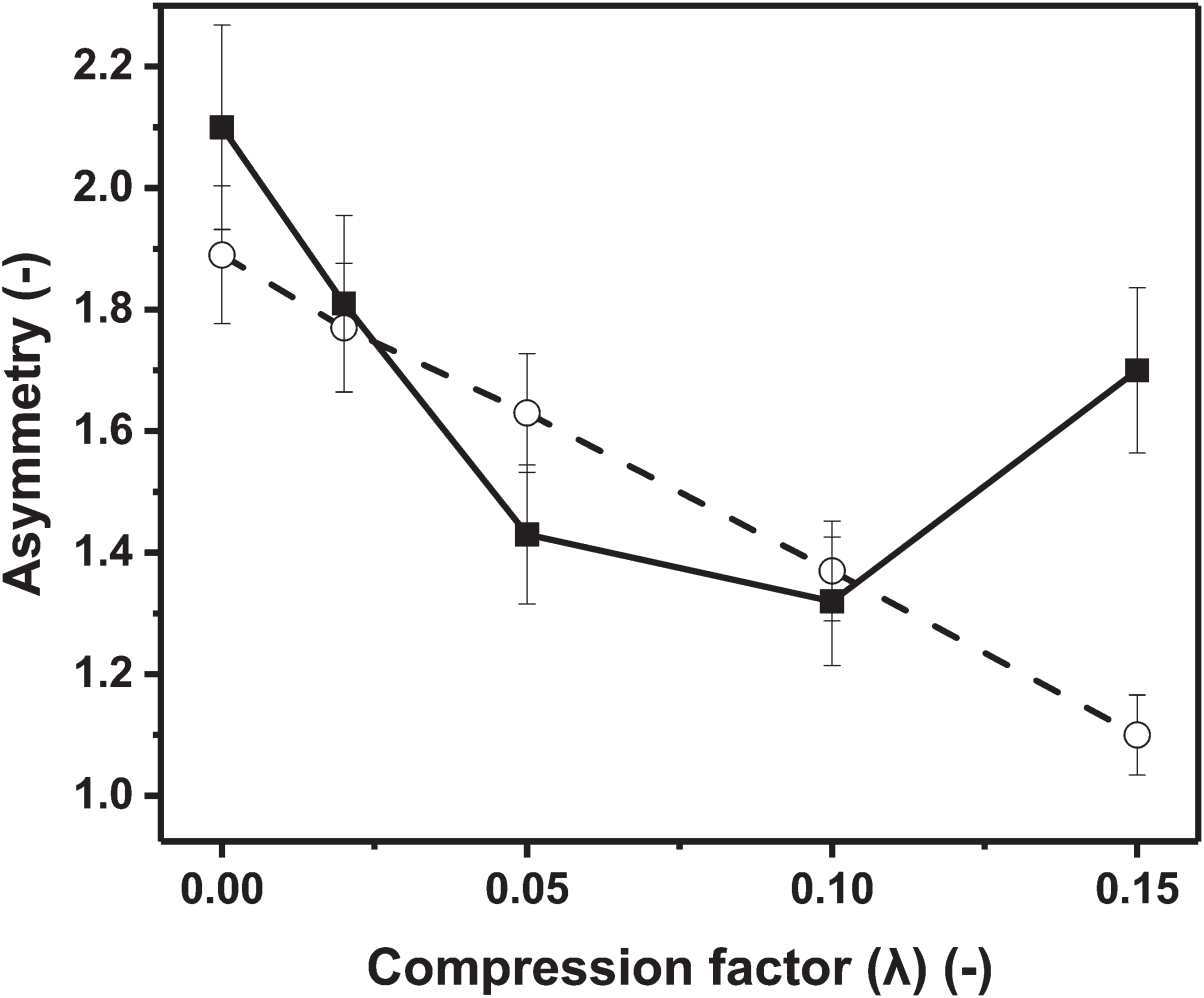
Impact of mechanical compression achieved by multiple incremental steps on asymmetry at bench (XK16) and pilot scale (BPG‐100/500) measured with 2% v/v acetone; (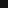) BPG 100/500; (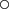) XK16.

The impact of mechanical bed compression at bench (XK16) and pilot scales (BPG100) was studied. The results are presented in Fig. 5 where changes in asymmetry at both bench and pilot scales were verified. At pilot scale the asymmetry reduced above a compression factor of 0.10. This was expected, as the bed diameter increases so the extent to which the column wall supports the bed material falls. This allows the longitudinal force down the column to increase.^3^ Exceeding a 0.10 compression factor created increasing levels of bed non‐uniformity. The degree of compression at the bottom of the column depends on the column diameter. Wider columns allow more compaction (less wall support effect).^7^, ^23^, ^24^, ^25^, ^26^ This highlights the fact that there can be no one size fits all approach to column packing across columns scales even when utilising the same chromatography matrix. At pilot scale, as the bed reached 0.10 compression factor, optimum asymmetry was achieved when mechanical compression by multiple incremental steps was applied.

## CONCLUSIONS

There is a need to understand the effect of the methods by which packed beds are compressed prior to operation. In particular, the impact of mechanical compression on column performance during scale‐up is poorly reported. This study aimed to investigate the impact on column efficiency when applying hydrodynamic and mechanical compression to beds formed from Sepharose CL‐6B.

Results showed better asymmetry and reduced plate height with increasing levels of mechanical compression, regardless of how this was applied (one step or multiple incremental steps). One step hydrodynamic compression followed a similar trend to mechanical compression with a lower plate height and an asymmetry closer to one. However multi‐step hydrodynamic compression caused flow instability, most likely due to the formation of regions of higher compaction towards the bottom of the packed bed which together resulted in poor column efficiency. With mechanical compression, an even distribution of pressure was applied from the top column diameter which gave better column efficiency as measured by both asymmetry and reduced plate height. The voidage decreased with compression, this would translate in smaller interparticle distances and consequently in increased mass transfer.

Mechanical compression by multiple incremental steps resulted in greater levels of product purity and yields than by mechanical compression with one step. The impact of mechanical bed compression during scale up was investigated, exceeding a 0.10 compression factor created increasing levels of bed non‐uniformity. Beyond a compression factor of 0.15, no further improvements in bed performance as measured by asymmetry or HETP were recorded for either of the methods of compression investigated.

We have shown column performance to be strongly influenced by the level of bed compression as well as the method by which compression is affected. Investigation of mechanical compression of different resins, such as ion exchange medium during adsorptive separations will form the basis of future work.
